# An online survey of encouragement strategies for older adults’ daily worries: a conversational robot versus a human partner

**DOI:** 10.3389/frobt.2026.1843519

**Published:** 2026-08-26

**Authors:** Lingxuan Xiang, Marin Nishimura, Hideaki Kikuchi

**Affiliations:** 1 Graduate School of Human Sciences, Waseda University, Saitama, Japan; 2 Faculty of Human Sciences, Waseda University, Saitama, Japan

**Keywords:** conversational robot, daily worries, emotional support, encouragement strategies, human–robot interaction, older adults

## Abstract

Conversational robots are increasingly proposed to provide everyday emotional support for older adults. However, it remains unclear which encouragement strategies are perceived as effective and whether interpersonal strategy preferences transfer to Human–Robot Interaction (HRI). This study compared the perceived effectiveness of five encouragement strategies when delivered by a conversational robot versus a human partner, examined moderation by worry type, and explored associations with recipients’ emotional states. Japanese young-old adults aged 65–74 (final N = 55) completed an online scenario-based survey. Participants were randomly assigned to a human or conversational robot condition, each combining an identity label with distinct voice characteristics (a human-recorded voice or a robot-recorded voice). They recalled one physical and one psychological daily worry and rated perceived encouragement for five strategy types: reassurance and affirmation, expressing concern, encouragement to act, offering specific actions, and distraction. Within-subject standardized ratings were analyzed using linear mixed-effects models with a participant random intercept. Positive and Negative Affect Schedule (PANAS) were examined exploratorily. Results showed that perceived effectiveness differed by strategy type and notably by condition. In the human condition, offering specific actions showed the highest relative effectiveness, with descriptively clearer differentiation among strategies. In the robot condition, reassurance and affirmation ranked highest, and differentiation among strategies was descriptively reduced. Distraction was consistently least preferred. The present study did not observe a statistically significant effect of worry type on the overall ranking patterns. Exploratory analyses suggested that positive affect was associated with encouragement evaluation at the raw-score level in the robot condition, whereas associations involving negative affect did not survive correction for multiple comparisons and are therefore interpreted descriptively rather than as statistically significant findings. These findings suggest that interpersonal encouragement strategy preferences may not directly transfer to human–robot interaction as operationalized in the present study. The observed differences between conditions may reflect the combined effect of the identity label and voice characteristics rather than the robot’s identity alone. The results highlight the need for partner-specific, context-adaptive encouragement design in conversational robots for older adults.

## 1 Introduction

Globally, population aging poses major challenges. In Japan, the aging rate has already reached 29.3% as of 2025 (Cabinet Office of Japan, 2025). Older adults often face daily physical and psychological worries, such as reduced physical strength, knee pain, and loneliness (Cabinet Office of Japan, 2020). Although these worries are generally not life-threatening, they can function as stressors associated with daily hassles (Lazarus and Folkman, 1984). In everyday life, people frequently share these worries in casual conversations, where listeners offer comfort and understanding as a fundamental part of social interaction (House et al., 1988; Cohen and Wills, 1985). However, older adults have fewer people to confide in (Cabinet Office of Japan, 2020). This highlights the need for more accessible ways for older adults to share their worries and receive emotional support in daily life.

As one approach to addressing the shortage of confidants among older adults, the demand for conversational agents is increasing (Matsuyama et al., 2010; Kamiyama et al., 2010; Nikkei, 2024). Conversational agents encompass a broad range of systems, including embodied conversational robots and voice- or text-based chatbots. Among these, this study focuses specifically on conversational robots, which combine verbal interaction with a perceived social presence that may be particularly relevant to emotional support contexts. Prior research suggests that everyday conversations with conversational agents (e.g., free talk, sharing positive experiences) can reduce psychological stress, alleviate loneliness, and enhance wellbeing among older adults (Wada and Shibata, 2007; Abd-Alrazaq et al., 2020). However, most studies evaluate emotional support as an overall outcome and rarely examine the specific linguistic strategies that agents use to provide it.

According to social support theory, support can be divided into several types, including emotional support (e.g., comfort and encouragement) and instrumental support (e.g., information and advice) (Cohen and Wills, 1985). Emotional support is particularly important because it helps reduce the psychological burden caused by daily hassles. Encouragement is a fundamental form of emotional support (House et al., 1988; Khan, 2013). It refers to utterances that aim to reduce the negative emotions of individuals experiencing worries. This study focuses on verbal encouragement, defining different utterances as categories of encouragement and perceived effectiveness as the extent to which participants report feeling encouraged. Prior research on human–human interaction suggests that encouragement is not a uniform construct; rather, its effectiveness depends on the interaction between contextual factors and strategy types (Ogawa, 2011; Ogawa, 2014; Ogawa and Nakazawa, 2014; see Section 2.2 for details). However, most of this evidence comes from human–human interaction, raising the question of whether these patterns hold when encouragement is provided by a conversational robot.

In HRI research, the CASA paradigm (Nass et al., 1994) and media equation theory (Reeves and Nass, 1996) suggest that users transfer social scripts from interpersonal contexts to interactions with agents. Based on this assumption, many studies argue that interpersonal support strategies can be directly applied to HRI (Abd-Alrazaq et al., 2020; Xiang et al., 2025). However, emerging evidence suggests that psychological processes in HRI may differ from those in interpersonal contexts (Hancock et al., 2011; Pan et al., 2025), raising the question of whether this assumption holds for encouragement strategies (see Section 2.2.2 for a detailed discussion). In addition, beyond the effects of partner type and worry type, recipients’ emotional states may also influence how they evaluate encouragement. Prior research suggests that emotional states shape evaluative judgments (Schwarz and Clore, 1983; Schwarz and Clore, 2003) and affect interaction outcomes with robots (Stock-Homburg, 2022). Yet, whether emotional states influence evaluations of specific encouragement strategies in HRI remains unexplored.

Based on the above analysis, this study identifies two key gaps. First, systematic comparisons of whether the same encouragement strategies produce similar effects when the conversation partner is a human or a conversational robot remain insufficient, particularly among older adults. Second, it remains unclear whether recipients’ emotional states are associated with evaluations of encouragement strategies, and whether such associations differ across human and robot contexts.

Based on these gaps, this study examines three main questions: whether the pattern of effective encouragement strategies differs between human and conversational robot partners; whether worry type moderates this pattern; and whether recipients’ emotional states are associated with strategy evaluations across the two partner conditions.

This study aims to make two contributions. Theoretically, it provides initial empirical evidence regarding the assumption that interpersonal support findings can be directly applied to HRI, thereby exploring the potential boundary conditions of emotional support theories in conversational robot contexts. Practically, the findings are expected to offer preliminary design considerations for selecting encouragement strategies in conversational robots for older adults.

To address these gaps, this study poses the following research questions.

RQ1: What is the relative effectiveness ranking of the five encouragement types in the context of older adults’ daily worries?

RQ2: Does this ranking differ by interaction partner (human vs. robot)?

RQ3: Does worry type (physical vs. psychological) alter this ranking? If so, does this pattern differ by interaction partner?

RQ4: Are older adults’ emotional states (positive and negative affect) associated with the evaluation of each encouragement strategy? Does this association vary by interaction partner (human vs. robot) and worry type?

## 2 Related work

### 2.1 Daily worries of older adults and emotional support from conversational robots

This study focuses on everyday worries of older adults rather than clinical psychological conditions. These worries can be categorized into physical aspects (e.g., health problems, sensory decline) and psychological aspects (e.g., loneliness, anxiety about the future, loss of social roles) (Cabinet Office of Japan, 2020).

Prior research has shown that conversational systems have potential to alleviate loneliness, provide emotional comfort, and promote psychological wellbeing among older adults (Matheus et al., 2025; Mehrabi and Ghezelbash, 2025). Such systems include companion-type social robots such as PARO (Wada and Shibata, 2007; Broekens et al., 2009; Yen et al., 2024), proactive social robots such as ElliQ (Broadbent et al., 2024), chatbots (Abd-Alrazaq et al., 2020), and dialogue systems powered by large language models (Treder et al., 2024; Abadir et al., 2024; Irfan et al., 2024). These technologies vary widely in their interaction modalities, ranging from non-verbal companionship to verbal interaction, and from scripted dialogue to open-ended conversation. However, these studies typically treat emotional support as a global outcome, leaving it unclear which specific verbal strategies account for perceived effectiveness. Among various forms of verbal support, encouragement is one of the most commonly observed responses in everyday conversation (House et al., 1988; Khan, 2013). Despite its prevalence, encouragement has not been systematically examined as an independent strategy in research on emotional support from conversational robots, leaving a gap in understanding which specific encouragement strategies are perceived as effective.

### 2.2 Encouragement strategies: from interpersonal contexts to HRI

#### 2.2.1 Definition and classification of encouragement

Encouraging utterances refer to verbal expressions aimed at alleviating negative emotions such as sadness, anxiety, dissatisfaction, or regret (Tanaka, 2015). Through a systematic analysis of encouragement scenarios, Tanaka (2015) proposed a five-type classification: reassurance and affirmation (Type A), expressing concern (Type B), encouragement to take action (Type C), offering specific actions (Type D), and distraction (Type E). The definitions and representative examples for each type are presented in Table 1.

**TABLE 1:** Classification of encouraging utterances and examples.

Encouragement type	Definition	Example
Type A: reassurance and affirmation	Affirm the other person’s situation or future with positive praise or reassurance	“It happens to everyone, so do not worry about it.” “You are amazing! I’m sure you can do it.”
Type B: expressing concern	Show sympathy or understanding by asking questions, indicating genuine concern	“That sounds tough. Are you okay?” “I see. You’re not pushing yourself too hard, are you?”
Type C: encouragement to take action	Motivate the person to act on their issues, though this can sometimes feel burdensome	“Why not give it a try first? It might help to experiment a little!” “It’s more enjoyable if you think of it lightly. Don’t take it so seriously.”
Type D: offering specific actions	Offer help, emphasizing assistance rather than doing it together	“If there’s anything I can do, I’ll help you out.” “I’m happy to help whenever you need.”
Type E: distraction	Shift the person’s focus away from their problems to relieve stress. Suggestions should be light and non-pressuring	“How about soaking in a nice warm bath?” “How about having a delicious dessert?”

#### 2.2.2 Transferring interpersonal theories to HRI

The CASA paradigm (Nass et al., 1994) and media equation theory (Reeves and Nass, 1996) suggest that users treat agents similarly to humans, transferring social scripts from interpersonal contexts. Based on this assumption, many studies have argued that emotional support strategies effective in interpersonal settings can be directly applied to HRI (Abd-Alrazaq et al., 2020). However, this assumption does not fully account for the distinct psychological processes that agents may elicit.

Three key differences are relevant. First, trust formation differs. Trust in humans develops through accumulated social interaction, whereas trust in agents relies more on functional reliability and physical design (Hancock et al., 2011). Second, intention interpretation differs. Users may expect follow-up actions from a human speaker but not from an agent, which may alter how they evaluate supportive utterances (Pan et al., 2025). Third, individual differences may be amplified. Social norms for interacting with agents are still being established, and factors such as age and cultural background may therefore exert a stronger influence on interaction outcomes than in interpersonal contexts (Heerink, 2011; Krämer et al., 2015).

These differences suggest that the same encouragement strategy type may produce different patterns of effectiveness depending on whether the speaker is a human or a robot. Directly comparing encouragement effectiveness between the two under controlled conditions is therefore necessary to examine the applicability of interpersonal findings to HRI. Empirical research in this direction remains limited.

#### 2.2.3 Moderation of encouragement effects by type of worries

The type of worries may also moderate encouragement effects. Physical worries (e.g., declining health, mobility limitations) typically involve clearer causal attributions and more specific directions for coping. In contrast, psychological worries (e.g., loneliness, loss of social roles) tend to be more abstract and diffuse, making them difficult to address through concrete solutions. The Optimal Matching Model (Cutrona and Russell, 1990) proposes that support effectiveness depends on the alignment between the type of support and the characteristics of the stressor. Preliminary evidence from interpersonal research suggests that the type of worries and its causal attribution are among the key factors influencing encouragement effectiveness (Ogawa, 2014). However, whether this pattern holds in HRI contexts, and whether it differs by conversational partner, remains unexplored.

### 2.3 Emotional state and the evaluation of encouragement

The emotional states of older adults fluctuate considerably in daily life, with age-related changes in emotion regulation shaping how they respond to social situations (Charles et al., 2001; Carstensen et al., 2003). The Affect-as-Information theory (Schwarz and Clore, 1983; Schwarz and Clore, 2003) suggests that individuals use their emotional experiences as information when making judgments. Positive emotions signal that the current situation is satisfactory, promoting heuristic processing and broader evaluation styles. Negative emotions signal that a problem exists, promoting systematic processing and more analytical evaluation (Schwarz, 2002).

This theory is also applicable to encouragement reception. Rather than passively absorbing encouraging messages, recipients actively evaluate whether a message fits their situation, whether it is sincere, and whether it is helpful (Goldsmith, 2004). Because these evaluations constitute a form of social judgment that draws on affective cues, the process is structurally consistent with the mechanism described in the theory. Based on this reasoning, individuals with higher positive affect may evaluate encouraging messages more leniently overall, while those with higher negative affect may evaluate them more cautiously, making finer distinctions across strategies.

However, the theory’s core predictions are based on general cognitive tasks, whereas encouragement reception also involves relational factors (Goldsmith, 2004). Moreover, in HRI, users’ emotional states have been shown to affect robot acceptance and interaction outcomes (Stock-Homburg, 2022; Spitale et al., 2023). Yet, the relationship between emotional state and evaluation of specific support strategies has not been systematically examined. In particular, it remains unclear whether this relationship varies by strategy type. Given that trust formation and intention interpretation differ between human and agent interactions (see Section 2.2.2), the role of emotional state in encouragement evaluation may also manifest differently in HRI contexts.

Accordingly, it remains unclear whether and how emotional states shape the evaluation of specific encouragement strategies, and whether such effects differ between human and robot interactions.

## 3 Materials and methods

### 3.1 Ethical statement

This study was approved by the Ethics Review Committee for Research Involving Human Subjects at Waseda University (Approval No. 2024-255). All participants provided informed consent online before taking part in the study. They were explicitly informed of the study’s purpose, procedures, and their right to withdraw at any time without providing a reason and without any adverse consequences. Given the vulnerability of the older adult population, participants who reported hearing, language comprehension, or production difficulties, as well as those currently experiencing or being treated for mental health conditions such as depression, severe anxiety, or cognitive impairment, were excluded during recruitment to minimize potential psychological risk. All collected data were anonymized, and no personally identifiable information was recorded. Data are stored securely on password-protected computers and will be disposed of appropriately after the retention period.

### 3.2 Experimental design

To examine encouragement effectiveness among older adults across conversational partners (human vs. conversational robot) and worry types (physical vs. psychological), this study employed an online scenario-based questionnaire, administered independently in a quiet environment with an estimated completion time of 30 min. To provide a clear and intuitive comparison for older adult participants, the conditions were labeled “robot” and “human.” In the robot condition, participants were told that their conversational partner was a robot, without specifying its physical form. The conversational partner manipulation combined a categorical label (human vs. robot) with a corresponding voice cue (see Section 3.4.3 for details). As a result, the two conditions differed in both social category and acoustic characteristics.

The experiment adopted a mixed design with one between-subjects factor (conversational partner: human vs. robot, randomly assigned) and two within-subjects factors (worry type: physical vs. psychological; encouraging utterance type: five types). Conversational partner was randomly assigned between subjects to prevent participants from inferring the study’s comparative purpose and to reduce task complexity, particularly for older adults. Worry type and encouragement type were presented in randomized order within subjects to control for individual differences in receptivity to encouragement, enabling more reliable comparisons across conditions.

Regarding the conversational scenario, both conditions were premised on imagining confiding daily worries to a close person and receiving encouragement. In the human condition, the confiding partner was set as a close friend. In the robot condition, the confiding partner was set as a conversational robot perceived as equally close. This close relationship setting aimed to capture natural encouragement in everyday contexts, avoiding potential tension from stranger interactions. Questionnaires were identical across conditions except for the conversational partner manipulation.

### 3.3 Participants

#### 3.3.1 Recruitment and inclusion criteria

In Japan, older adults are defined as those aged 65 years or above; however, this age range encompasses considerable variation in cognitive function, physical condition, and ability to use devices. To enhance sample homogeneity, participants were limited to Japanese native speakers aged 65–74 (young-old), with no gender restrictions. Participants were recruited via Japanese crowdsourcing platforms (CrowdWorks and Lancers). All participants were required to complete online questionnaires using a computer, smartphone, or tablet and to comprehend the textual and auditory materials presented. Family members were permitted to provide technical assistance with device operation without interfering with their responses.

Participants were excluded if they met any of the following criteria: (1) hearing, language comprehension, or production impairments; (2) current treatment for or suspicion of mental disorders (e.g., depression, cognitive impairment); (3) severe anxiety or psychological distress; or (4) suicidal ideation. These criteria were established for two reasons. First, the experiment involved the presentation of auditory materials and the imagination of troubling scenarios; hearing or language impairments may affect full comprehension of the experimental materials. Second, experimental tasks centered on confiding about troubles may impose a burden on individuals with high psychological vulnerability, and they were therefore excluded on ethical grounds.

During recruitment, eligible participants were informed of the age range and exclusion criteria in advance. Only those who confirmed that they met the inclusion criteria and provided consent proceeded to complete the questionnaire.

#### 3.3.2 Sample size

An *a priori* power analysis using G*Power 3 (Faul et al., 2007) with α = 0.05, 1 − β = 0.80, and a medium effect size (f = 0.25) indicated a minimum of approximately 50 participants. Accounting for potential attrition, incomplete responses, or insufficient data quality in studies involving older adults, 62 participants were recruited (human condition: 31 (21 male, 10 female); robot condition: 31 (19 male, 12 female)).

#### 3.3.3 Final sample

After data screening for completeness and data quality (see Section 3.7), the final sample comprised 55 participants (human condition: 28 (21 male, 7 female); robot condition: 27 (15 male, 12 female)). The mean age was 67.3 years (SD = 2.53). Demographic details are presented in Table 2.

**TABLE 2:** Participant demographics by condition.

Characteristic	Human (*n* = 28)	Robot (*n* = 27)	Overall (*N* = 55)
Age *M* (SD)	67.2 (2.26)	67.4 (2.82)	67.3 (2.53)
Male/female	21/7	15/12	36/19
PA *M* (SD)	25.18 (5.46)	25.89 (6.14)	—
NA *M* (SD)	14.79 (6.13)	14.15 (4.93)	—

PA, positive affect; NA, negative affect (Japanese version of PANAS; Sato and Yasuda, 2001).

### 3.4 Experimental materials

All experimental materials were reviewed by three native Japanese speakers prior to the main experiment. Materials approved by all three were accepted. If any concerns were raised, the three reviewers discussed and revised the material until consensus was reached. Revisions followed shared principles: natural Japanese, consistent politeness level (desu/masu style with a slightly casual tone), and restriction to a single category per item.

#### 3.4.1 Worry scenario materials

Worry scenarios were categorized into physical and psychological based on a Cabinet Office of Japan report (2020). Physical worries encompass health conditions (e.g., decline in physical strength, chronic illness), mobility difficulties (e.g., difficulty climbing stairs), sensory changes (e.g., vision decline), fatigue (e.g., becoming easily fatigued), and difficulties in daily living (e.g., increased burden of housework). Psychological worries encompass loneliness and social isolation (e.g., reduced communication with family), anxiety about the future (e.g., worry about health deterioration), loss of social roles (e.g., diminished sense of presence in society), generational gaps (e.g., differences in values with younger generations), and a sense of unstructured time (e.g., feeling that days are monotonous). Two to three representative examples were created for each category to help older participants understand the distinction. Validity checks confirmed that each scenario expressed a single worry type in natural Japanese. The procedure by which participants engaged with these scenarios is described in Section 3.6. Examples of physical worries included “My knees hurt, making stairs difficult” and “My lower back hurts.” Examples of psychological worries included “Everyday conversations with family have decreased” and “Connections with society have diminished since retiring.”

#### 3.4.2 Encouraging utterance materials

Encouraging utterances were constructed based on Tanaka’s (2015) five-type classification (see Section 2.2.1): reassurance and affirmation (Type A), expressing concern (Type B), encouragement to take action (Type C), offering specific actions (Type D), and distraction (Type E). A typological framework was adopted because this study focuses on functional differences among strategies rather than specific wording. Two representative example utterances were created for each type to aid participant understanding, with consistency maintained in sentence length, tone, and everyday comprehensibility to minimize the influence of surface linguistic features on evaluations. Validity checks confirmed that each utterance belonged to the intended type, did not conflate other types, and felt appropriate as encouragement in response to a worry. In the experiment, each type was presented with its definition, supplementary explanations, and two example utterances. The definitions and examples after validity confirmation are presented in Table 1.

#### 3.4.3 Audio materials

The example utterances of encouragement were presented in both text and audio. The main reason for adopting this format was that older adult participants might gradually forget the conversational partner condition during the lengthy questionnaire process when relying solely on text. The inclusion of audio helped to continuously reinforce participants’ awareness of whether they were in the human or robot condition.

The audio for the human condition was recorded by a female native Japanese speaker in her 20s (a graduate student). The audio for the robot condition was generated using the speech synthesis of RoBoHoN (Sharp Corporation). Both audio conditions were intended to provide participants with clear cues about the conversational partner. A single human speaker was used to control for the influence of individual voice characteristics on evaluations. RoBoHoN was chosen because, as a conversational robot widely recognized in Japan, its synthesized voice is highly distinguishable in a Japanese-language context.

Given that acoustic features of the voice (e.g., perceived gender and age) might influence evaluations, the questionnaire explicitly instructed participants to evaluate the encouraging utterances based solely on their content, disregarding voice characteristics. A post-questionnaire item assessed whether voice characteristics had influenced their ratings (see Section 3.6).

**FIGURE 1:**
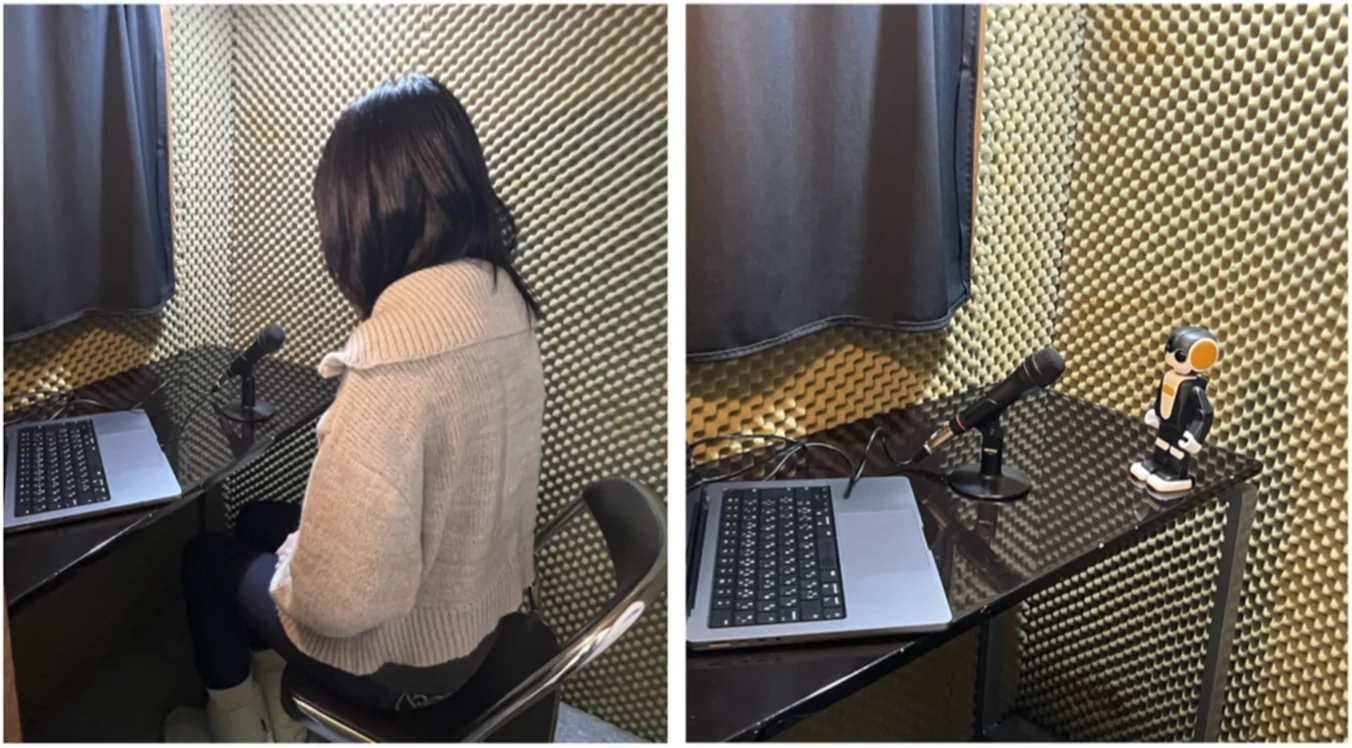
Recording setup for audio materials.

All audio materials were recorded in a soundproofed room using a condenser microphone (Sony ECM-PCV80U). Recording and basic editing were performed using GarageBand (Apple Inc.). The recording setup is shown in Figure 1. Prior to the main experiment, 10 native Japanese-speaking university students verified all audio materials. The evaluation criteria included: (1) whether the pronunciation conformed to standard Japanese; (2) whether any parts were inaudible; and (3) whether the audio felt appropriate as an encouraging utterance. Materials that did not pass verification were re-recorded and re-verified. After verification, noise reduction and volume normalization were applied to minimize the influence of recording quality differences on evaluations.

### 3.5 Measures

#### 3.5.1 Encouragement effectiveness

The primary dependent variable was participants’ subjective rating of the extent to which they felt encouraged by each encouraging utterance, measured using a 0–100 Visual Analogue Scale (VAS; Shirahama et al., 2021), where 0 indicated “not encouraged at all” and 100 indicated “extremely encouraged.” Participants provided their ratings by moving a slider on a continuous scale. The VAS was chosen over Likert scales to avoid central tendency bias, and over direct numerical entry to prevent favoritism toward specific numbers (e.g., multiples of 10), yielding more granular data for detecting subtle differences among strategies (Shirahama et al., 2021).

#### 3.5.2 Emotional state (PANAS)

Participants’ emotional states were measured using the 16-item Japanese version of the PANAS (Sato and Yasuda, 2001), adapted from the original 20-item PANAS (Watson et al., 1988). The scale consists of eight Positive Affect (PA) items (active, proud, strong, enthusiastic, determined, excited, alert, inspired) and eight Negative Affect (NA) items (scared, afraid, upset, nervous, jittery, distressed, ashamed, irritable). PA and NA represent two independent dimensions rather than opposite ends of a single continuum. Each item was rated on a 6-point Likert scale (1 = “not at all” to 6 = “very much”), and scores were summed separately for PA and NA.

Participants rated their emotional states over the past 24 h to capture recent emotional context that might influence evaluations, while avoiding potential interference from transient states at the time of the experiment.

### 3.6 Procedure

#### 3.6.1 Step 1: pre-questionnaire

Participants first completed basic demographic items (age, gender), followed by the PANAS. The PANAS was administered prior to the rating tasks to capture emotional states before exposure to experimental stimuli. In the robot condition, participants also reported their frequency of interaction with conversational robots (often/occasionally/never).

#### 3.6.2 Step 2: recall and articulation of worries

To ground evaluations in personal experience, participants recalled one physical worry and one psychological worry before providing ratings. Brief definitions and examples of the two worry types were presented (see Section 3.4.1). Participants then orally articulated each worry based on their own experience and transcribed the content into the questionnaire. Oral articulation was included to enhance immersion in the conversational scenario. Text recording ensured that participants referred to the same worry context during subsequent evaluations. No audio recordings were made during this step. Participants were instructed to: (1) articulate a single worry without conflating multiple worries; (2) describe the content, background, and situation as specifically as possible; (3) skip topics they were reluctant to discuss. The order of the two worry types was randomized to control for order effects.

#### 3.6.3 Step 3: encouragement presentation and rating

For each worry type, the definitions, supplementary explanations, and two example utterances for each of the five encouragement types were presented sequentially in both text and audio (see Sections 3.4.2, 3.4.3). In the human condition, human-recorded audio was used; in the robot condition, RoBoHoN-recorded audio was used. After reading and listening to each encouragement type, participants immediately rated its effectiveness using the VAS slider (0–100). Participants were instructed to: (1) imagine a casual conversation in which they share their worries and receive encouragement; (2) evaluate based on their understanding of the definition of each encouragement type; (3) note that the presented utterances are examples of strategies, not specific responses to their particular worry; (4) avoid being influenced by the specific wording of the examples; (5) provide ratings intuitively.

Steps 2 and 3 were conducted separately for each worry type. Each participant completed 5 encouragement types × 2 worry types = 10 ratings. The presentation order of the five encouragement types was randomized to control for order effects.

#### 3.6.4 Step 4: post-questionnaire

After completing all ratings, participants filled out a post-questionnaire covering three areas. First, a manipulation check confirmed whether participants’ perception of the experimental condition was consistent with the intended manipulation (i.e., whether they correctly identified the audio source as robot voice/human voice/unsure), the voice’s perceived gender (male/female/unsure), and the voice’s perceived age (young/middle-aged/older/unsure). Second, an assessment of voice influence examined whether voice characteristics biased effectiveness ratings. Participants reported whether they were able to focus on evaluating content without being influenced by voice characteristics (completely able/slightly influenced/basically unable), and whether voice characteristics actually influenced their ratings (not at all/slightly/considerably). Third, participants provided feedback on their experimental experience (e.g., whether it was interesting, easy to understand, or tiring) and free-response comments. In the human condition, an additional item asked whether participants had previously heard the voice used in the experiment, to rule out familiarity effects.

### 3.7 Data preprocessing and statistical analysis

#### 3.7.1 Data screening and preprocessing

Data were screened based on predefined criteria. Participants were excluded if they did not complete all rating tasks, had missing responses for core items, or had extremely short (<5 min) or long (>3 h) completion times. Recorded worry content was also reviewed, and data were excluded if the content clearly did not correspond to the assigned worry type. Within-subject standardization (z-score transformation) was applied to encouragement effectiveness ratings to control for individual differences in scale usage. This transformation emphasizes relative evaluation patterns across strategies within each participant and enhances comparability across participants.

#### 3.7.2 Primary analysis (RQ1–RQ3)

A linear mixed-effects model (LMM) was used to account for the repeated measures structure, with participants as a random intercept. Standardized encouragement effectiveness scores served as the dependent variable. The main effects of conversational partner, worry type, and encouragement type, along with their two-way and three-way interactions, were entered as fixed effects. Type A (Reassurance and Affirmation) served as the reference category for encouragement type. RQ1 was examined through the main effect of encouragement type. RQ2 was examined through the interaction between conversational partner and encouragement type. RQ3 was examined through the interaction between worry type and encouragement type, and through the three-way interaction among conversational partner, worry type, and encouragement type. When a main effect or interaction reached statistical significance, *post hoc* comparisons were conducted using estimated marginal means with Tukey adjustment. As a supplementary robustness check, the primary LMM was re-estimated with self-reported voice influence included as an additional fixed effect. Voice influence was derived from the post-questionnaire item asking whether voice characteristics actually influenced ratings (not at all/slightly/considerably) and was dichotomized as “not at all” versus “slightly” or “considerably,” along with its interaction with encouragement type.

#### 3.7.3 Exploratory analysis (RQ4)

To explore whether participants’ emotional states were associated with evaluations of each encouragement strategy, Pearson correlation analyses were conducted between PA and NA scores and the scores for each encouragement type, separately for each condition (conversational partner × worry type, resulting in four conditions). The analysis was conducted at two levels. The first level used raw scores (0–100) to examine whether emotional state was associated with the absolute level of perceived effectiveness for each strategy. The second level used within-subject standardized scores. Standardization eliminates individual differences in scale usage (see Section 3.7.1), allowing examination of whether emotional state altered the relative ranking of strategy preferences within each participant, in other words, whether the influence of emotional state was strategy-specific. This two-level approach distinguishes between two types of effects: non-strategy-specific effects (overall shifts in receptivity across strategies) and strategy-specific effects (selective shifts in preferences for particular strategies).

#### 3.7.4 Post-questionnaire analysis

Descriptive statistics were calculated for the manipulation check items, voice influence assessment, and experimental feedback to confirm condition identification, assess potential bias from voice characteristics, and inform future design improvements.

All statistical analyses were performed using R (version 4.5.3).

## 4 Results

### 4.1 Primary analysis (RQ1–RQ3)

A linear mixed-effects model was conducted as described in Section 3.7.2. Table 3 presents the raw VAS means and standard deviations for the five encouragement strategies under each condition, providing information on absolute perceived effectiveness. Because the primary analyses were conducted on within-subject standardized scores, the EMM rankings reported below reflect within-participant relative preferences rather than absolute VAS ratings. Fixed effects were tested using Type III ANOVA with Satterthwaite’s approximation. The full model results are presented in Table 4.

**TABLE 3:** Raw VAS means and standard deviations for encouragement effectiveness by condition.

Condition	Type A M (SD)	Type B M (SD)	Type C M (SD)	Type D M (SD)	Type E M (SD)
Human–physical (*n* = 28)	67.18 (23.40)	71.46 (18.69)	66.96 (17.14)	73.11 (21.70)	62.32 (20.50)
Human–psychological (*n* = 28)	63.86 (25.21)	67.25 (22.72)	64.68 (20.19)	70.46 (23.07)	59.68 (18.04)
Human overall (*N* = 56)	65.52 (24.16)	69.36 (20.72)	65.82 (18.59)	71.79 (22.23)	61.00 (19.18)
Robot–physical (*n* = 27)	70.07 (23.17)	74.96 (13.97)	64.59 (21.95)	67.56 (22.25)	68.26 (18.17)
Robot–psychological (*n* = 27)	74.04 (13.78)	68.67 (19.27)	69.74 (17.68)	75.19 (15.51)	65.74 (19.32)
Robot overall (*N* = 54)	72.06 (18.99)	71.81 (16.97)	67.17 (19.91)	71.37 (19.39)	67.00 (18.62)
Overall (*N* = 110)	68.73 (21.92)	70.56 (18.93)	66.48 (19.17)	71.58 (20.79)	63.95 (19.06)

Values represent raw VAS, scores (0–100). “Human Overall” and “Robot Overall” combine scores across both worry types within each condition; “Overall” combines scores across all conditions and worry types. Type A, reassurance and affirmation; Type B, expressing concern; Type C, encouragement to take action; Type D, offering specific actions; Type E, distraction.

**TABLE 4:** Type III ANOVA results for the full and condition-specific models.

Panel	Effect	*Num*DF	*Den*DF	*F*	*p*	η_p_^2^
Overall model	Partner	1	530	0.000	1.000	0.000
	Worry	1	530	0.001	0.974	0.000
	Type	4	530	7.566	<0.001***	0.054
	Partner × worry	1	530	1.212	0.272	0.002
	Partner × type	4	530	2.796	0.026*	0.021
	Worry × type	4	530	1.531	0.192	0.011
	Partner × worry × type	4	530	0.920	0.452	0.007
Human condition	Worry	1	270	0.662	0.417	—
	Type	4	270	7.023	<0.001***	0.094
	Worry × type	4	270	0.246	0.912	0.004
Robot condition	Worry	1	260	0.554	0.457	—
	Type	4	260	3.438	0.009**	0.043
	Worry × type	4	260	2.150	0.075^†^	0.032

***p < 0.001, **p < 0.01, *p < 0.05, ^†^ *p* < 0.10.

#### 4.1.1 RQ1: overall differences among the five encouragement strategies

**FIGURE 2:**
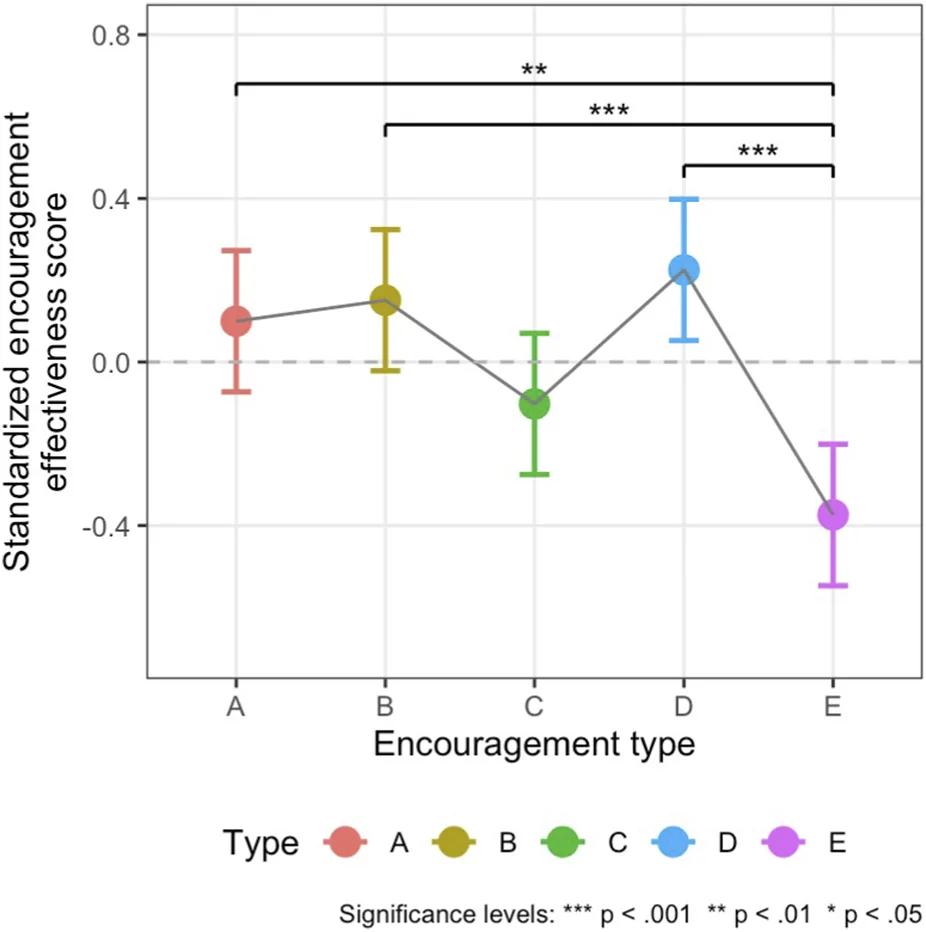
Estimated marginal means of encouragement effectiveness by strategy type.

The main effect of encouragement type was statistically significant (F(4, 530) = 7.566, p < 0.001, ηp^2^ = 0.054). As shown in Figure 2, the overall EMM ranking was Type D > Type B > Type A > Type C > Type E. Tukey *post hoc* comparisons revealed that Types D, B, and A were each statistically significantly higher than Type E (all p < 0.01). No other pairwise differences reached statistical significance. These findings indicate that the manner of encouragement is an important factor influencing perceived effectiveness.

#### 4.1.2 RQ2: influence of conversational partner

**FIGURE 3:**
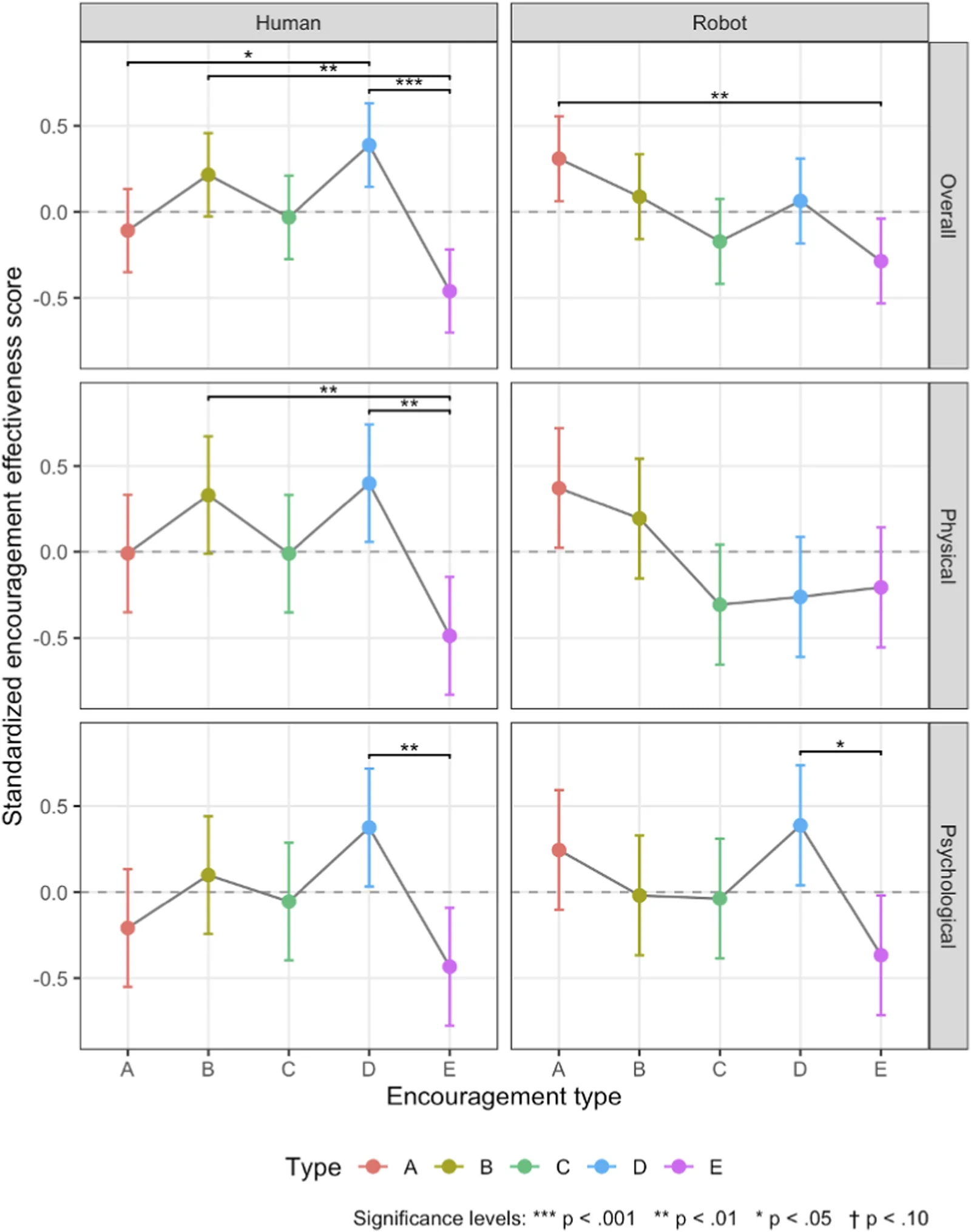
Estimated marginal means of encouragement effectiveness by strategy type, partner condition, and worry type.

The interaction between conversational partner and encouragement type was statistically significant, F(4, 530) = 2.796, p = 0.026, ηp^2^ = 0.021 (Table 4), indicating that the relative effectiveness of encouragement strategies differed between the human and robot conditions. We therefore examined simple effects within each partner condition (Figure 3).

In the human condition, the simple main effect of encouragement type was statistically significant, F(4, 270) = 7.023, p < 0.001, ηp^2^ = 0.094 (Table 4). Estimated marginal means (EMMs) indicated the following ranking: Type D (0.388) > Type B (0.215) > Type C (−0.033) > Type A (−0.109) > Type E (−0.461). Tukey-adjusted *post hoc* comparisons showed that Type D was rated higher than Type A (p = 0.036) and Type E (p < 0.0001), and Type B was rated higher than Type E (p = 0.001). The worry type × encouragement type interaction was not statistically significant (p = 0.912), suggesting that the human-condition ranking was stable across physical and psychological worries.

In the robot condition, the simple main effect of encouragement type was also statistically significant, F(4, 260) = 3.438, p = 0.009, ηp^2^ = 0.043 (Table 4), but the pattern differed from the human condition. The EMM ranking was Type A (0.309) > Type B (0.088) > Type D (0.063) > Type C (−0.173) > Type E (−0.287). Only the contrast between Type A and Type E reached statistical significance (p = 0.009). The worry type × encouragement type interaction showed a marginal trend (p = 0.075), driven primarily by Type D shifting from fourth (physical worries) to first (psychological worries) within the robot condition.

Taken together, three descriptive differences characterized the partner contrast. First, Type D was the highest-ranked strategy in the human condition but showed reduced stability in the robot condition, whereas Type A ranked highest in the robot condition but was comparatively lower in the human condition. Second, strategy differentiation was descriptively larger in the human condition (ηp^2^ = 0.094) than in the robot condition (ηp^2^ = 0.043). Because these values come from separate simple-effect models with different error structures, this contrast should be interpreted cautiously; nevertheless, the pattern is consistent with weaker separation among strategy evaluations in the robot condition. Third, Type E was consistently lowest or near-lowest across both partner conditions, suggesting that it was generally the least preferred strategy regardless of partner.

##### 4.1.2.1 Supplementary robustness check

To assess whether the partner differences were substantially attributable to self-reported voice influence, we added voice influence (dichotomized as “not at all” vs. “slightly” or “considerably”) to the primary LMM as an additional fixed effect, including its interaction with encouragement type (see Section 3.7.2). The partner × type interaction remained substantively unchanged. Notably, the partner × Type D interaction, which was the largest contributor to the overall interaction, was identical in both models (b = −1.042, p = 0.003). The main effect of voice influence was not statistically significant (b = −0.118, p = 0.558), and its interactions with encouragement type were not statistically significant (all p > 0.05), except for a small interaction with Type E (b = 0.681, p = 0.017). These results suggest that the observed partner-specific pattern in encouragement effectiveness was not primarily explained by self-reported voice influence, while acknowledging the limitations of self-report and the reduced precision associated with small subsamples. Full results are reported in Table 5.

**TABLE 5:** Robustness check for self-reported voice influence.

	Original model			Robustness model		
Term	*b*	SE	*p*	*b*	SE	*p*
Panel A: partner × type interaction						
Partner (robot) × type B	−0.517	0.352	0.141	−0.517	0.351	0.140
Partner (robot) × type C	−0.678	0.352	0.054^†^	−0.685	0.351	0.051^†^
Partner (robot) × type D	−1.042	0.352	0.003**	−1.042	0.351	0.003**
Partner (robot) × type E	−0.099	0.352	0.778	−0.043	0.351	0.902
Panel B: voice influence terms (robustness model only)						
Voice influence (main)	—	—	—	−0.118	0.202	0.558
Voice influence × type B	—	—	—	−0.005	0.285	0.986
Voice influence × type C	—	—	—	−0.086	0.285	0.763
Voice influence × type D	—	—	—	0.001	0.285	0.998
Voice influence × type E	—	—	—	0.681	0.285	0.017*

***p < 0.001, **p < 0.01, *p < 0.05, ^†^ *p* < 0.10. Voice influence was dichotomized: 0 = “not at all” (*n* = 14), 1 = “slightly” or “considerably” (*n* = 41). Panel A compares the partner × encouragement type interaction coefficients between the original model and the robustness model. Panel B shows the voice influence terms in the robustness model only. The dash (—) indicates that the term was not included in the original model.

#### 4.1.3 RQ3: influence of worry type

The present study did not observe a statistically significant interaction between worry type and encouragement type (F(4, 530) = 1.531, p = 0.192, ηp^2^ = 0.011), nor a statistically significant three-way interaction (F(4, 530) = 0.920, p = 0.452, ηp^2^ = 0.007; Table 4). At the overall sample level, the present study did not observe a statistically significant effect of worry type on the effectiveness ranking of the five encouragement strategies.

Because the overall interaction was not statistically significant, condition-specific patterns should be interpreted with caution and treated as exploratory observations rather than confirmed effects. With this caveat, the conditional analyses reported in Section 4.1.2 revealed descriptively different patterns between conditions. In the human condition, the interaction between worry type and encouragement type was clearly not statistically significant (p = 0.912, ηp^2^ = 0.004), whereas in the robot condition, a marginal trend was observed (p = 0.075, ηp^2^ = 0.032). This trend was primarily driven by Type D, whose EMM shifted from the physical worry condition (−0.262) to the psychological worry condition (0.388). While this pattern cannot be considered a confirmed moderating effect given the statistically non-significant overall interaction, it may suggest a tentative direction for future investigation with larger samples.

### 4.2 RQ4: emotional state

Pearson correlation analyses were conducted at two levels (raw scores and within-subject standardized scores) as described in Section 3.7.3. All correlation coefficients are presented in Table 6. Given the exploratory nature of RQ4, uncorrected p-values are reported as descriptive evidence, with Benjamini–Hochberg FDR correction applied as a robustness check (q-values reported in Table 6). After correction, three PA correlations in the robot condition remained statistically significant at the raw-score level (q < 0.05): PA with Type E in the robot–physical worry condition, and PA with Types A and E in the robot–psychological worry condition. At the standardized-score level, two NA correlations were observed in the robot condition, but neither survived FDR correction (robot–physical NA × Type C, q = 0.280; robot–psychological NA × Type E, q = 0.240). These associations are therefore interpreted descriptively rather than as statistically significant findings. These results underscore the exploratory nature of the affect-related findings.

**TABLE 6:** Pearson correlations between emotional state and encouragement effectiveness ratings with Benjamini–Hochberg FDR-adjusted q values.

		Type A		Type B		Type C		Type D		Type E	
Condition	Affect	*r*	*q*	*r*	*q*	*r*	*q*	*r*	*q*	*r*	*q*
Raw scores											
Human–physical	PA	0.168	0.604	0.382*	0.228	0.433*	0.172	0.380*	0.228	0.286	0.360
	NA	−0.045	0.820	−0.225	0.500	0.034	0.909	−0.091	0.806	−0.021	0.939
Human–psych	PA	0.172	0.604	0.205	0.536	0.315	0.316	0.215	0.517	0.325^†^	0.316
	NA	−0.233	0.500	−0.151	0.658	−0.114	0.765	−0.228	0.500	−0.097	0.804
Robot–physical	PA	0.399*	0.228	0.379^†^	0.228	0.456*	0.169	0.321	0.316	0.668***^a^	0.004
	NA	−0.148	0.659	0.079	0.843	−0.256	0.464	−0.113	0.765	−0.036	0.909
Robot–psych	PA	0.555**^a^	0.036	0.200	0.550	0.322	0.316	0.289	0.360	0.608***^a^	0.016
	NA	−0.010	0.959	0.057	0.909	−0.046	0.909	−0.176	0.604	0.290	0.360
Standardized scores											
Human–physical	PA	—	—	—	—	—	—	—	—	—	—
	NA	—	—	—	—	—	—	—	—	—	—
Human–psych	PA	—	—	—	—	—	—	—	—	—	—
	NA	—	—	—	—	—	—	—	—	—	—
Robot–physical	PA	—	—	—	—	—	—	—	—	—	—
	NA	—	—	—	—	−0.511**	0.280	—	—	—	—
Robot–psych	PA	—	—	—	—	—	—	—	—	—	—
	NA	—	—	—	—	—	—	—	—	0.475*	0.240

*N* = 28 (human condition), *N* = 27 (robot condition). PA, positive affect; NA, Negative affect. *r* = Pearson correlation coefficient (uncorrected). *q* = Benjamini–Hochberg FDR-adjusted *p* value, computed separately for raw scores (40 tests) and standardized scores (40 tests). ^†^ *p* < 0.10, *p < 0.05, **p < 0.01, ***p < 0.001 (uncorrected).

^a^ Statistically significant after FDR, correction (*q* < 0.05). “—” indicates not statistically significant (*p* > 0.10). For standardized scores, neither statistically significant correlation survived FDR, correction.

#### 4.2.1 Raw score level

Positive affect showed broad positive correlations with effectiveness ratings across multiple strategies. In the human–physical worry condition, PA was statistically significantly correlated with Types B, C, and D. In the robot–physical worry condition, PA was statistically significantly correlated with Types A, C, and E, with Type B showing a marginal trend. In the robot–psychological worry condition, PA was statistically significantly correlated with Types A and E. After FDR correction, three PA correlations remained statistically significant at the raw-score level: PA with Type E in the robot–physical worry condition (r = 0.668, q = 0.004), PA with Type A in the robot–psychological worry condition (r = 0.555, q = 0.036), and PA with Type E in the robot–psychological worry condition (r = 0.608, q = 0.016). This pattern suggests that PA may reflect an overall elevation in encouragement evaluation rather than selective preferences for specific strategies. Negative affect showed no statistically significant correlations with any strategy type at the raw score level in any condition.

#### 4.2.2 Standardized score level

After standardization, all correlations with PA that had been observed at the raw-score level were no longer evident, confirming that PA was associated primarily with a global elevation in ratings without altering the relative preference ordering among strategies (a non-strategy-specific effect). In contrast, two strategy-specific patterns emerged for NA in the robot condition at the uncorrected level. In the robot–physical worry condition, NA showed a negative correlation with Type C (r = −0.511, uncorrected p = 0.007, q = 0.280). In the robot–psychological worry condition, NA showed a positive correlation with Type E (r = 0.475, uncorrected p = 0.012, q = 0.240). These correlations involving negative affect did not survive FDR correction and are therefore interpreted descriptively rather than as statistically significant findings. No comparable patterns were observed in the human condition at this level. Notably, the strategy-specific patterns for NA were observed only in the robot condition. Given the small sample sizes per condition (n = 27–28), these findings should be interpreted with caution and require replication with larger samples.

### 4.3 Post-questionnaire results

#### 4.3.1 Manipulation check

In the human condition, all 28 participants (100%) correctly identified the voice source as a human voice and the voice gender as female. Regarding voice age, 25 participants (89.3%) judged it as young, and 3 (10.7%) as middle-aged. All 28 participants reported that they had not heard the voice outside of the study context. In the robot condition, all 27 participants (100%) correctly identified the voice source as a robot voice. Regarding voice gender, 16 (59.3%) judged it as male, 7 (25.9%) as female, and 4 (14.8%) responded unsure. Regarding voice age, 26 (96.3%) judged it as young, and 1 (3.7%) responded unsure. These results confirm that the conversational partner manipulation was effective.

#### 4.3.2 Voice influence

Regarding whether participants were able to focus on evaluating content without being influenced by voice characteristics, in the human condition, 16 participants (57.1%) responded “completely able,” and 12 (42.9%) responded “slightly influenced”; in the robot condition, the numbers were 15 (55.6%) and 12 (44.4%), respectively. Regarding whether voice characteristics actually influenced their ratings, in the human condition, 6 participants (21.4%) responded “not at all,” 21 (75.0%) responded “slightly,” and 1 (3.6%) responded “considerably”; in the robot condition, the numbers were 8 (29.6%), 18 (66.7%), and 1 (3.7%), respectively. These results indicate that the majority of participants reported voice characteristics had some degree of influence on their evaluations, with most indicating “slight” influence. This suggests that voice characteristics may have introduced some contextual interference despite instructions to focus on content. In the robot condition, 26 participants (96.3%) reported never having conversed with a conversational robot, and 1 (3.7%) responded “occasionally.” This indicates that evaluations were made with minimal prior experience with conversational robots.

#### 4.3.3 Participant feedback

Free-response comments covered several themes. Several participants reported feeling encouraged or comforted during the experiment; in the human condition, some noted that having a friend listen made them feel happy, while in the robot condition, one participant mentioned that receiving encouragement from a robot felt easier than talking to a person. Some participants indicated that verbally articulating their worries prompted self-reflection, helping them reassess their situation. Attitudes toward robots varied: some expressed positive expectations for future applications, describing the experience as novel and enjoyable, whereas others expressed more cautious views, noting that despite advances in technology, they still found it difficult to imagine a robot as someone to confide in. Some participants also offered suggestions for improvement, including more response patterns and options for voice gender and age. Regarding audio materials, one participant suggested adding more prosody to the encouraging voice, while another noted that the robot’s childlike voice felt reassuring.

## 5 Discussion

This study systematically compared the perceived effectiveness of five encouragement strategies among young-old adults aged 65–74 under both human and robot conditions using an online scenario-based design. The results showed that the relative effectiveness pattern varied statistically significantly by conversational partner (conversational partner × encouragement type interaction: p = 0.026), and that the influence of emotional state on strategy evaluation was context-dependent. These findings suggest that, under the present label-plus-voice manipulation, interpersonal support strategies may not produce identical effectiveness patterns in HRI contexts. However, because the manipulation combined social category labels with distinct acoustic characteristics, the relative contribution of each factor remains to be disentangled in future research. Accordingly, the condition-specific patterns discussed below should be interpreted as reflecting the combined effect of the identity label and voice characteristics, rather than the robot’s identity alone. The following sections discuss the key findings, theoretical implications, practical implications, limitations, directions for future research, and conclusion.

### 5.1 Key findings

#### 5.1.1 Overall effectiveness and moderation by conversational partner (RQ1 and RQ2)

In the overall analysis, Types A (Reassurance and affirmation), B (Expressing concern), and D (Offering specific actions) each showed statistically significantly higher effectiveness than Type E (Distraction), whereas differences among Types A, B, and D did not reach statistical significance. This indicates that encouragement is not a unitary behavior; its effectiveness varies by strategy type, consistent with interpersonal findings (Ogawa, 2011; Ogawa, 2014; Ogawa and Nakazawa, 2014). However, because the ranking differed statistically significantly between conditions (RQ2), the following discussion focuses on condition-specific patterns.

##### 5.1.1.1 Type D and type B in the human condition

Type D consistently ranked highest across both worry types in the human condition (physical M = 0.399, psychological M = 0.376), with clear differentiation among strategies (ηp^2^ = 0.094). This is partially consistent with the Optimal Matching Model (Cutrona and Russell, 1990), which predicts that action-oriented support is effective for controllable problems. However, Type D also ranked highest for psychological worries, extending beyond this prediction. One *post hoc* interpretation is that utterances such as “I can help you” may function not merely as commitments to action, but as signals of relational investment. For older adults, whose social networks tend to shrink with age (Carstensen, 1992; Carstensen et al., 2003), such messages may convey relational value regardless of whether the worry is practically solvable. In the human condition, participants could reasonably expect the speaker to fulfill such commitments, which may have lent credibility to Type D. Type B also performed relatively well in the physical worry condition (M = 0.330), but its effectiveness decreased in the psychological worry condition (M = 0.099). This may be because physical worries are relatively concrete and observable, and the empathetic concern expressed in Type B may align with the recipient’s need to be acknowledged (Goldsmith, 2004). In contrast, psychological worries are more abstract and diffuse, and simple expressions of concern may be perceived as insufficient.

##### 5.1.1.2 Type A in the robot condition

In the robot condition, Type A replaced Type D as the highest-ranked strategy (M = 0.309), and overall differentiation among strategies was reduced (ηp^2^ = 0.043). Type A offers general comfort through affirmation and reassurance without presupposing the speaker’s ability to act or deeply understand the recipient’s experience. For a robot, which tends to be perceived as capable but limited in social depth (de Graaf et al., 2015), this communicative style may align well with users’ expectations. The reduced differentiation among strategies may reflect attenuated sensitivity to strategy differences when the encouragement is perceived as coming from a robot. In addition, the absence of established social norms for robots (Krämer et al., 2015) may leave users without a clear framework for evaluating robot encouragement, further contributing to reduced differentiation.

##### 5.1.1.3 Instability of type D across conditions

Type D was consistently effective in the human condition and in the robot–psychological worry condition (M = 0.388), but dropped to fourth place in the robot–physical worry condition (M = −0.262). This variation may reflect the degree of match between the capability implied by the utterance and the perceived capability of the conversational partner, although perceived capability was not directly measured in this study. For physical worries, participants’ expectations of help were concrete and practical. When a robot offered to help, participants may have recognized its limitations in taking physical action, reducing perceived encouragement. This is consistent with findings on intention interpretation in HRI (Hancock et al., 2011; Pan et al., 2025). For psychological worries, which do not carry expectations of practical action, Type D may have been interpreted as symbolic support rather than a literal commitment to act. Whether participants reinterpreted Type D in this way was not directly measured and requires future validation.

##### 5.1.1.4 Consistently low effectiveness of type E

Type E (Distraction) ranked lowest across nearly all conditions. Many of the worries presented in this study, such as chronic pain and reduced social contact, are often persistent in nature and not easily set aside (Charles et al., 2001). In such contexts, distraction may be perceived as avoidance or a lack of genuine attention to the worry. This contrasts with the mechanism underlying Type D’s ineffectiveness in the robot–physical condition: whereas Type D may have failed due to perceived capability limitations, Type E may fail because it signals reluctance to engage with the worry. Thus, Type D and Type E may fail for fundamentally different reasons: Type D fails when there is a mismatch between implied and perceived capability, whereas Type E fails because it signals disengagement from the worry itself.

#### 5.1.2 Influence of worry type (RQ3)

In the overall model, the present study did not observe a statistically significant interaction between worry type and encouragement type (p = 0.192), nor a statistically significant three-way interaction (p = 0.452). In the human condition, the interaction was clearly not statistically significant (p = 0.912), whereas in the robot condition, a marginal trend emerged (p = 0.075). As discussed in Section 5.1.1, this trend was primarily driven by Type D, whose effectiveness in the robot condition appeared to depend on whether the worry context evoked expectations of practical action. These findings tentatively suggest that the influence of worry type may not operate uniformly across strategies, but may be more pronounced for strategies in which perceived capability alignment is salient. However, the effect size of the key partner × type interaction was very small (ηp^2^ = 0.021), and the current sample may have lacked sufficient power to detect such subtle higher-order effects; larger samples are needed to confirm this possibility.

#### 5.1.3 Emotional state and encouragement evaluation (RQ4)

Exploratory correlation analyses at the raw score and standardized score levels revealed qualitatively distinct patterns for PA and NA.

PA exhibited a non-strategy-specific effect. At the raw score level, PA showed broad positive correlations with multiple strategies, but these correlations disappeared entirely after standardization. This indicates that PA may globally elevate encouragement evaluations without altering the relative preference ordering among strategies. This pattern is consistent with the Affect-as-Information theory, which predicts that positive affect facilitates heuristic processing (Schwarz, 2002), leading to broadly elevated evaluations without fine distinctions among strategies.

NA, in contrast, exhibited a strategy-specific pattern at the uncorrected level. At the raw score level, NA showed no statistically significant correlations with any strategy. After standardization, however, two correlations emerged in the robot condition only at the uncorrected level: a negative correlation with Type C (Encouragement to take action) in the physical worry condition (r = −0.511, uncorrected p = 0.007, q =0 .280), and a positive correlation with Type E (Distraction) in the psychological worry condition (r = 0.475, uncorrected p = 0.012, q =0.240). Neither correlation survived FDR correction; these associations are therefore interpreted descriptively rather than as statistically significant findings. Although these associations should be interpreted with caution, the pattern is broadly consistent with the idea that negative affect may facilitate more differentiated processing of encouragement strategies. The negative correlation with Type C may reflect reduced receptivity to action-oriented prompts under elevated negative affect. The positive correlation with Type E may reflect a preference for temporary disengagement under heightened distress.

These strategy-specific patterns for NA emerged only in the robot condition. One possible explanation is that in the human condition, evaluations may be more strongly shaped by stable relational judgments such as closeness and trust (Reis and Clark, 2013), which may buffer the influence of transient emotional states. In the robot condition, where the framework for relational judgments is weaker (Krämer et al., 2015), the influence of emotional state as background information (Schwarz and Clore, 2003) may become more salient. This pattern may suggest that stable relational judgments in human interaction attenuate the influence of transient emotional states, a possibility that warrants further investigation. Stock-Homburg (2022) similarly noted that emotional states influence interaction evaluations with robots; the present findings tentatively suggest that this influence may be more observable in HRI contexts under the present design.

Given the small sample sizes per condition (n = 27–28), these findings are preliminary. Their consistency with the Affect-as-Information theory reflects *post hoc* interpretation rather than *a priori* hypothesis testing, and replication with larger samples using confirmatory designs is needed.

### 5.2 Implications

#### 5.2.1 Theoretical implications

This study provides conditional empirical evidence regarding the applicability of the CASA paradigm (Nass et al., 1994; Nass and Moon, 2000) in emotional support. Although users exhibited social responses to the robot, the relative preference patterns did not simply replicate those in interpersonal contexts. The variation in Type D’s effectiveness across conditions suggests that users may evaluate encouragement strategies based on the match between the capability implied by the utterance and the perceived capability of the conversational partner, indicating that the CASA effect in emotional support may be conditional and strategy-dependent rather than uniform. However, because the partner manipulation confounded the identity label with voice characteristics, the extent to which these differences are attributable to the robot’s social category *per se* remains to be established.

The findings also speak to the Optimal Matching Model (Cutrona and Russell, 1990), which proposes that support effectiveness depends on the match between support type and stressor characteristics. The present results suggest that even when such a match appears to exist, a strategy may still be less effective if the provider is perceived as lacking the capability to enact the implied support. This raises the possibility that perceived capability of the support provider may constitute an additional factor worth incorporating into the model, although this interpretation remains speculative and awaits future validation given that perceived capability was not directly measured in this study.

The exploratory findings regarding emotional state provide preliminary evidence for extending the Affect-as-Information theory (Schwarz and Clore, 1983; 2003) to the context of encouragement reception. The non-strategy-specific association between positive affect and encouragement evaluation and the strategy-specific patterns involving negative affect are broadly consistent with the theory’s predictions concerning heuristic and systematic processing. Nevertheless, the correlations involving negative affect did not survive FDR correction and are therefore interpreted descriptively rather than as statistically significant findings. These results tentatively suggest that emotional states may shape not only global evaluations of encouragement but also the relative differentiation among support strategies. However, these correspondences are based on exploratory analyses and require confirmation through larger samples and confirmatory designs.

Methodologically, this study offers one of the first controlled comparisons of encouragement strategy effectiveness between human and robot speakers among older adults. Many studies on emotional support from conversational robots implicitly assume that interpersonal support strategies can be transferred to HRI contexts. The statistically significant interaction between conversational partner and encouragement type observed in this study suggests that such transfer cannot be assumed, although the confounded manipulation means that the observed differences may partly reflect voice characteristics rather than the robot’s social category alone. This finding highlights the value of direct comparative designs in identifying the boundary conditions of interpersonal support theories within HRI.

#### 5.2.2 Practical implications

The present findings offer several design considerations for encouragement functions in conversational robots for older adults.

First, for default strategy selection, Type A demonstrated the most stable relative performance in the robot condition based on the standardized analyses. When a conversational system cannot reliably infer the user’s type of worry or emotional state, adopting Type A strategies that provide general reassurance without implying concrete action may serve as a relatively safe default option, provided that this pattern is confirmed in real conversational interactions. Such strategies appear less dependent on assumptions about the robot’s capability and may reduce the risk of perceived mismatch.

Second, for context-sensitive strategy adaptation, the differential performance of Type D across worry types suggests that adaptive adjustment may be warranted. Type D was relatively effective for psychological worries but less effective for physical worries in the robot context. When systems can identify whether a user’s worry involves concrete physical issues or more abstract psychological worries, adjusting encouragement strategies accordingly may enhance perceived effectiveness. For physical worries, strategies such as Type A or Type B may be more appropriate, whereas Type D may be considered in psychological contexts where expectations of literal action are lower.

Third, regarding emotional sensitivity, users with higher levels of negative affect may respond less favorably to action-oriented prompting in robot contexts. If conversational systems incorporate affect detection mechanisms, limiting action-oriented prompting when elevated negative affect is detected, and instead using more supportive strategies such as Type A or Type B, may improve user experience. However, because the associations between negative affect and strategy-specific evaluation did not survive correction for multiple comparisons, this suggestion requires confirmation in future studies.

In addition, because 96.3% of participants in the robot condition reported no previous interaction experience with conversational robots, the findings underlying these implications may partly reflect initial reactions shaped by novelty effects or media-driven stereotypes rather than evaluations based on mature or sustained interaction experience. Given the scenario-based design, relatively small subsamples, and the exploratory nature of the emotional state findings, these practical implications should be extended with caution.

### 5.3 Limitations

This study has several limitations that should be considered when interpreting the findings.

First, the sample was limited to young-old adults aged 65–74 in Japan, recruited through crowdsourcing platforms. The preference for indirect expression and emotional restraint characteristic of Japanese culture (Markus and Kitayama, 1991) may have been particularly favorable to strategies such as Type A, warranting caution when generalizing to other cultural contexts. Participants capable of using crowdsourcing platforms may also possess higher digital literacy than the broader population within their age group. The relatively small sample sizes per condition (n = 27–28) limited statistical power, particularly for interaction effects and the exploratory emotional state analyses. The *a priori* power analysis was based on a medium effect size (f = 0.25), but the observed effect size for the key interaction between conversational partner and encouragement type (ηp^2^ = 0.021) was substantially smaller. This suggests that the study may have been underpowered for detecting the interaction effects central to RQ2 and RQ3, and that larger samples are needed to establish the robustness of these findings. In addition, the relatively small sample size did not permit examination of potential gender differences in encouragement preferences, and the gender distribution differed somewhat between conditions (human: 21 male, 7 female; robot: 15 male, 12 female). Whether gender moderates the effectiveness of encouragement strategies in HRI remains an open question.

Second, the conversational partner manipulation in this study combined a categorical label (human vs. robot) with a corresponding voice cue (human-recorded voice vs. RoBoHoN-recorded voice). This bundled design means that the observed differences between conditions may reflect not only the social category of the conversational partner but also differences in acoustic characteristics such as naturalness, perceived gender, perceived age, and vocal quality. Approximately 70% of participants reported that voice characteristics somewhat influenced their evaluations (human: 75.0%; robot: 66.7%). Although the supplementary robustness check (Section 4.2.2) indicated that the key interaction remained stable after controlling for self-reported voice influence, self-report measures may not fully capture the extent of acoustic influence on evaluations. Future studies should disentangle label and voice effects, for example, by crossing partner labels with voice types in a factorial design, to determine the relative contribution of each factor.

Third, participants evaluated encouragement through recall and imagination rather than receiving it in real-time conversation. Social presence and contextual authenticity may have been weaker than in face-to-face interactions. In the robot condition, 96.3% of participants reported never having conversed with a conversational robot, severely limiting the ecological validity of the robot-condition findings; their evaluations may have been shaped primarily by novelty effects or media-driven stereotypes rather than by responses grounded in mature interaction experience. Furthermore, participants in the robot condition were asked to imagine a robot perceived as equally close as a friend, which may have been psychologically difficult given their near-total absence of prior robot interaction experience, potentially producing evaluations based on unrealistic expectations rather than relatable experience.

Fourth, evaluations were based on single presentations. Trust building and relationship development are dynamic processes (Hancock et al., 2011), and the present design cannot address whether the relative effectiveness of strategies changes over sustained interactions.

Fifth, only auditory stimuli were presented without visual information. The physical presence of an embodied robot may evoke stronger social presence (Lee et al., 2006) but may also introduce factors such as the uncanny valley effect (Mori et al., 2012). The present findings reflect encouragement effectiveness under voice-only conditions, and their applicability to multimodal interactions remains to be confirmed.

Sixth, emotional state was measured using the PANAS with a 24-h retrospective window rather than assessing momentary affect at the time of evaluation. While this choice was made to avoid potential interference from the experimental task itself, it reduces the temporal precision of the association between emotional state and encouragement evaluation. The exploratory findings regarding emotional state should therefore be interpreted with this measurement limitation in mind.

### 5.4 Future directions

Several directions for future research emerge from these findings and their limitations.

A key priority is to examine the psychological mechanisms underlying the observed partner differences. Future studies should directly measure participants’ perceptions of the conversational partner along dimensions such as perceived empathic ability, action capability, and trustworthiness, and examine whether these perceptions mediate the interaction between conversational partner and encouragement type. Utterance interpretation tasks or think-aloud methods could verify whether participants reinterpret Type D utterances as symbolic support rather than literal action commitments. In addition, the finding that NA’s strategy-specific effects emerged only in the robot condition suggests that stable relational judgments may attenuate the influence of emotional state in interpersonal contexts. Future research could test this possibility by manipulating relationship closeness to further clarify differences between human–robot and human–human emotional support.

The present design also highlights the need for methodological extension. Future studies should employ diverse voice stimuli and extend to multimodal interactions that incorporate visual presentation, examining whether the appearance and expressions of embodied robots further influence strategy effectiveness. Findings from scenario-based designs should also be validated in real conversational contexts. In addition, longitudinal designs should investigate whether effectiveness patterns change as user–robot relationships develop over repeated interactions. Whether the relative advantage of Type A reflects initial expectations that shift as trust increases is a question worthy of investigation.

A third priority concerns participant diversity. Larger samples incorporating individual difference variables such as gender, personality traits, technology acceptance, and prior robot experience would allow re-examination of the exploratory emotional state findings with confirmatory designs. Cross-cultural comparisons would help distinguish between culturally specific and generalizable mechanisms.

Finally, these findings may inform encouragement strategies design in conversational systems powered by large language models. When such systems can identify users’ worry types and infer emotional states, they may dynamically select strategies based on the conditional effectiveness patterns observed in this study. Advancing research along these directions may contribute to a more refined theoretical framework of encouragement strategies in HRI and support evidence-based design of emotional support systems for older adults.

### 5.5 Conclusion

This study used an online scenario-based survey to compare the perceived effectiveness of five encouragement strategies delivered by a conversational robot versus a human partner among Japanese young-old adults (aged 65–74). The findings revealed that the relative effectiveness of encouragement strategies differed notably by condition. Specifically, in the human condition, offering specific actions tended to be evaluated most positively, whereas in the robot condition, reassurance and affirmation was generally preferred, with weaker differentiation among strategies overall. Distraction was consistently the least preferred across conditions. The present study did not observe a statistically significant effect of worry type on the overall ranking patterns, although the robot condition suggested possible context-dependent variation. Additional analyses further suggested that emotional state may influence encouragement evaluation differently in human–robot interaction. In particular, positive affect produced a non-strategy-specific global elevation in ratings, whereas negative affect was associated with strategy-specific patterns in the robot condition only at the uncorrected level; these associations are interpreted descriptively rather than as statistically significant findings. Taken together, these findings suggest that encouragement strategies effective in interpersonal communication may not directly transfer to human–robot interaction as operationalized in the present study. The observed differences between conditions may reflect the combined effect of the identity label and voice characteristics rather than the robot’s identity alone. The results highlight the importance of developing partner-specific and context-adaptive encouragement strategies for conversational robots designed to support older adults emotionally.

## Data Availability

The raw data supporting the conclusions of this article will be made available by the authors, without undue reservation.
